# Surface Quality and Environmental Impact Analysis of Ball Burnishing on Al8090 Aluminum–Lithium Alloy

**DOI:** 10.3390/ma18061252

**Published:** 2025-03-12

**Authors:** Suleyman Cinar Cagan

**Affiliations:** Department of Mechanical Engineering, Mersin University, Ciftlikkoy, Mersin 33343, Türkiye; cinarcagan@mersin.edu.tr

**Keywords:** ball burnishing, Al8090 aluminum–lithium alloy, surface quality, advanced manufacturing techniques, power consumption, carbon emission, sustainability

## Abstract

This study investigates the optimization of the ball burnishing process for Al8090 aluminum–lithium alloy, focusing on surface quality, mechanical properties, and sustainability metrics. A mixed-design L_18_ Taguchi experimental approach was employed to evaluate the effects of three critical parameters: burnishing force, feed rate, and number of passes under two lubrication conditions—dry and minimum quantity lubrication (MQL). Surface roughness, Brinell hardness, power and energy consumption, and carbon emissions were measured to assess technical and environmental performance. The results revealed that the MQL environment significantly improved surface roughness, achieving the lowest Ra value of 0.562 µm with a force of 200 N, a feed rate of 0.05 mm/rev, and four passes. In contrast, the highest Brinell hardness (43.6 HB) was observed in dry conditions with a force of 100 N, a feed rate of 0.1 mm/rev, and two passes. Energy consumption and carbon emissions were minimized in the MQL condition, with the lowest energy consumption recorded as 0.0169 kWh and corresponding carbon emissions of 0.0084 kg CO_2_. These findings highlight the trade-offs between surface quality, hardness, and sustainability, providing valuable insights for optimizing the ball burnishing process for advanced materials like Al8090.

## 1. Introduction

Aluminum alloys have long been favored in aerospace, automotive, and defense industries due to their low density, high specific strength, and excellent corrosion resistance [1]. Among these, aluminum–lithium (Al-Li) alloys, such as Al8090, stand out due to the unique advantages of lithium addition, including reduced weight, improved stiffness, and enhanced fatigue performance. These properties make Al8090 particularly suitable for high-performance and weight-sensitive applications, such as aircraft structures and space vehicles [2,3]. However, like many high-performance materials, the surface quality of Al8090 plays a critical role in its overall mechanical performance, wear resistance, and fatigue life. Surface defects or suboptimal surface properties can significantly compromise the material’s reliability, especially in demanding service conditions. Enhancing surface properties is, therefore, essential for ensuring long-term reliability and optimal performance in such applications [4].

Despite their exceptional mechanical properties, aluminum–lithium alloys, including Al8090, face challenges in aerospace applications due to their environmental sensitivity, such as susceptibility to stress corrosion cracking and exfoliation corrosion [2,5]. These issues can compromise the material’s longevity and negate surface enhancements achieved through processes like burnishing.

Aerospace aluminum components often undergo anodization to mitigate environmental effects [6]. However, anodization introduces surface defects that can reduce durability and serve as initiation sites for fatigue cracks under cyclic loading [7]. Additionally, fastener holes, typically drilled after anodization, remain untreated and cannot benefit from burnishing, making them critical for structural integrity [8,9].

Ball burnishing offers a promising solution by improving the surface integrity and mechanical properties of Al8090, addressing some of these challenges and enhancing its performance in demanding applications. Ball burnishing is an effective cold-working surface finishing process that has been widely recognized for its ability to improve surface integrity without material removal [10]. The process involves the plastic deformation of the surface layer by rolling a hardened ball over the workpiece under controlled force, which reduces surface roughness and induces compressive residual stresses that can enhance fatigue strength and wear resistance [11,12]. This dual benefit of surface smoothing and mechanical strengthening has made ball burnishing a popular choice for improving the performance of critical components. Although numerous studies have demonstrated the effectiveness of ball burnishing in enhancing surface quality and performance for materials like stainless steel, titanium alloys, and superalloys [13,14,15], the impact of this process on aluminum alloys such as Al8090 has yet to be thoroughly explored. This lack of research is particularly significant given the growing emphasis on optimizing surface finishing processes for advanced materials like Al8090.

The process parameters of ball burnishing, such as the applied force, feed rate, and number of passes, significantly influence the final surface characteristics [16]. Moreover, the choice of lubrication environment during burnishing, ranging from conventional dry conditions to environmentally conscious MQL, has emerged as a critical factor affecting surface quality, energy consumption, and overall process sustainability [17]. Dry burnishing offers simplicity and cost benefits, yet MQL has gained attention for its potential to reduce environmental impact while maintaining or even enhancing process performance [18]. In particular, MQL aligns with the growing emphasis on sustainable manufacturing practices, balancing process efficiency and environmental responsibility. In today’s manufacturing landscape, where energy efficiency and reduced carbon footprints are increasingly important, integrating sustainability metrics such as power consumption, energy usage, and carbon emission calculations into evaluating surface finishing processes is imperative [19,20].

Recent advancements in sustainable manufacturing have emphasized integrating eco-friendly techniques into machining and surface finishing processes. Studies have shown that hybrid nanofluid-assisted minimum quantity lubrication methods significantly enhance machining performance by reducing cutting forces, surface roughness, and energy consumption while lowering carbon emissions [21,22]. MQL system has demonstrated superior lubrication and cooling properties, leading to improved surface quality and reduced ability to wear in various materials, including aluminum alloys [23,24]. Additionally, the adoption of cooling techniques has proven effective in minimizing cutting temperatures and improving tool life, particularly when combined with MQL in hybrid systems [25,26]. These methods enhance process efficiency and align with sustainability goals by reducing the environmental impact of machining operations [27]. Furthermore, sustainability assessments in machining processes have highlighted the critical role of energy consumption and carbon emissions as key metrics for evaluating the environmental trade-offs of different lubrication and cooling strategies [22]. This underscores the importance of exploring how these advanced lubrication/cooling techniques can be adapted to optimize the performance of Al8090.

Despite the significant body of research on ball burnishing for various materials, there remains a clear gap in the literature regarding the systematic investigation of burnishing parameters on Al-Li alloyed Al8090, especially under varying lubrication strategies. To address this gap, the present study focuses on optimizing the ball burnishing process for Al8090 with a particular emphasis on both surface quality and sustainability metrics, adopting a mixed design L_18_ Taguchi experimental approach to systematically evaluate the effects of three critical parameters—burnishing force, feed rate, and number of passes—each at three distinct levels. In addition to traditional surface integrity measures such as surface roughness (Ra and Rz) and Brinell hardness, this study uniquely incorporates assessments of power and energy consumption as well as carbon emissions, thereby providing a comprehensive evaluation of both process performance and environmental impact. This holistic approach not only addresses the technical aspects of surface quality but also aligns with the broader goals of sustainable manufacturing by quantifying the environmental trade-offs associated with different process environments; this contributes to the growing demand for eco-friendly manufacturing solutions and offers valuable insights into the trade-offs between surface quality and process efficiency.

## 2. Materials and Methods

### 2.1. Workpiece Material

The study utilized a solid rod with a diameter of 20 mm and a length of 500 mm. The rod was divided into 40 mm intervals for each experiment, and the ball burnishing process was applied to these sections. The chemical composition and physical properties of Al8090 aluminum–lithium alloy are detailed in Table 1. In addition to aluminum (94.7%) as the main component, the alloy contains primary alloying elements such as lithium (2.5%), copper (1.3%), and magnesium (0.9%). This composition gives the alloy superior mechanical properties, including a tensile strength of 450 MPa and yield strength of 370 MPa. The alloy’s Brinell hardness value of 105 and elongation at a break of 7% demonstrate suitable mechanical properties for industrial applications. At the same time, its low density of 2.54 g/cm³ offers a significant weight advantage compared to standard aluminum alloys. This combination of properties makes Al8090 alloy ideal for applications requiring high performance and low weight, particularly in the aerospace and aviation industries.

### 2.2. Experimental Setup

**Figure 1:**
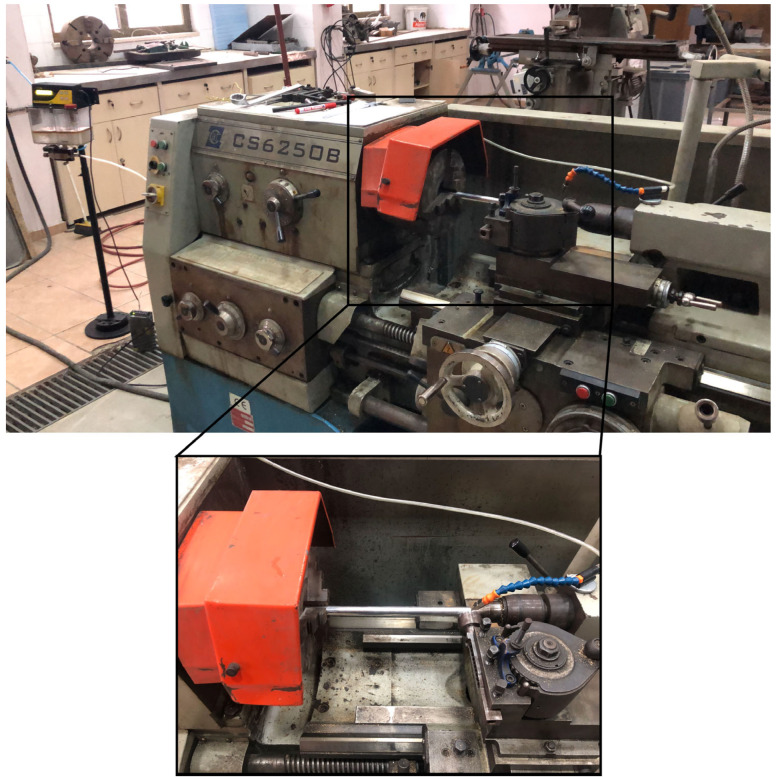
The schematic view of the experimental setup.

**Figure 2:**
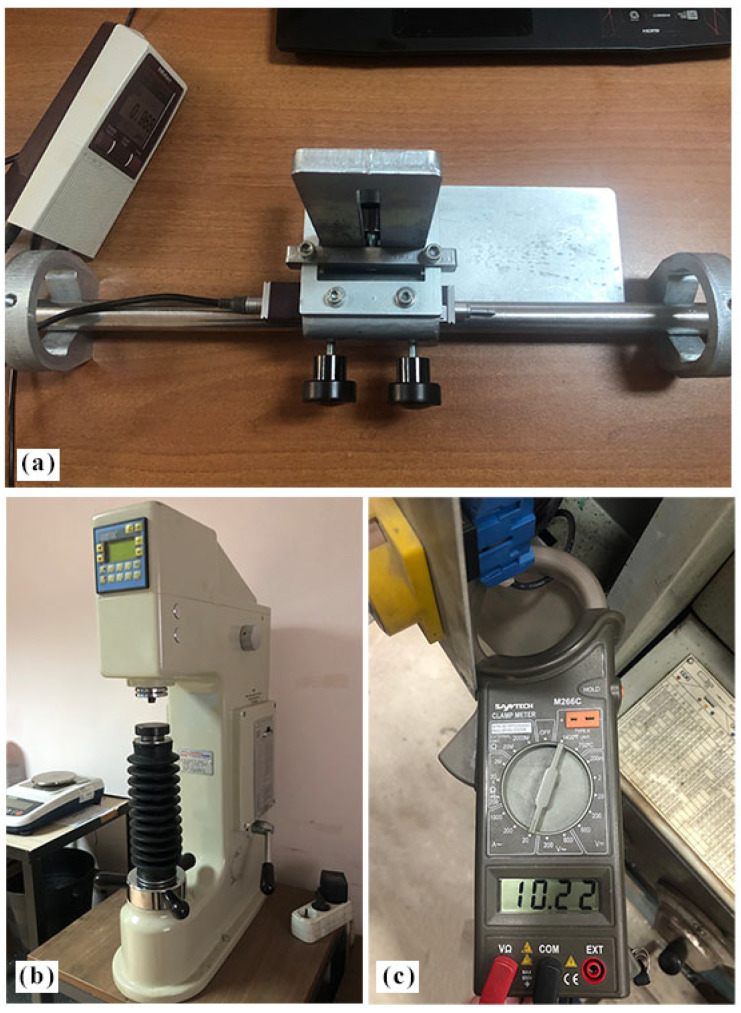
Pictorial view of the devices used for measurement: (**a**) surface roughness, (**b**) hardness, and (**c**) clamp meter.

The experimental setup comprised a conventional lathe machine (Baoji Machine Tool Group, CS6250B, Baoji, China) with a custom-engineered ball burnishing tool holder (Figure 1). The burnishing ability incorporated an AISI 52100 steel ball (10 mm diameter) with a hardness of approximately 700 HV and a surface roughness of 0.01 µm, mounted on a versatile holder designed to facilitate dry and MQL burnishing conditions. The ability holder used in this study is a custom-designed model developed and manufactured in our own workshop. The strategic selection of tool material with superior hardness characteristics enabled optimal crush strength relative to the workpiece, allowing precise control over surface roughness modification through regulated feed rates and burnishing forces. Force progression was continuously monitored via an integrated force gauge throughout the process. A systematic cleaning routine was maintained to preserve surface integrity and prevent complex particle contamination at the tool-specimen interface, thereby avoiding potential surface damage from abrasive particles. Precise workpiece alignment and stability were achieved through a dual-support configuration utilizing a three-jaw chuck and tailstock center arrangement. Figure 2 shows the pictorial view for surface roughness, hardness, and clamp meter.

An MQL system was integrated into the experimental setup as the primary cooling mechanism, aligning with modern sustainable manufacturing principles. The system was precisely calibrated to deliver cooling fluid at 5 bar pressure, with each spray pulse dispensing 0.0036 mL of lubricant at a frequency of 2 Hz. This optimized configuration achieved an economical fluid consumption rate of 25.92 mL/hour. The MQL nozzle targeted the critical contact zone between the burnishing tool and the workpiece interface to ensure maximum cooling and lubrication efficiency.

During the ball burnishing process, CUTTEX 22-R (Belgin Oil, Kocaeli, Türkiye), a high-performance metalworking fluid, was applied using the MQL method. This specialized lubricant is formulated with highly refined base oils and advanced performance additives. The fluid exhibits a kinematic viscosity of 27.5 cSt at 40 °C and a flash point of 220 °C. It contains engineered anti-wear and extreme-pressure (EP) additives, forming a robust protective film layer under high temperature and pressure conditions. The EP additives react chemically with the metal surface to create a highly durable lubricating film that maintains its integrity even under severe operating conditions, effectively reducing friction and facilitating heat dissipation. The lubricant’s advanced formulation ensures excellent surface finish quality and dimensional accuracy while preventing tool wear during burnishing.

### 2.3. Process Parameters and Experimental Design

The experimental investigation was designed to assess the impact of various process parameters on surface quality enhancement. Four important process parameters were identified as crucial control factors: burnishing conditions (C), force (F), feed rate (Fr), and number of passes (P). The burnishing force levels ranged from 50 N to 200 N and were carefully chosen to ensure ideal surface deformation. Feed rate values ranging from 0.05 to 0.2 mm/rev were used to evaluate the impacts of different processing speeds, and the number of passes was adjusted from 1 to 4 to examine the combined impact of repeated processing. In addition, three different burnishing conditions (dry and MQL) were used to evaluate the influence of lubricating conditions. The specific levels for each process parameter are presented in Table 2 [28]. The test parameters were determined through a literature review and preliminary trials to simulate industrial burnishing conditions while aligning with the capabilities of the custom-designed tool holder and experimental setup.

The experiments were conducted as single trials due to the high reliability and repeatability of the setup. The custom-designed tool holder and controlled conditions ensured consistent results. While additional repetitions could enhance statistical validation, the primary objective was to examine the effects of the selected parameters under controlled conditions.

**Figure 3:**
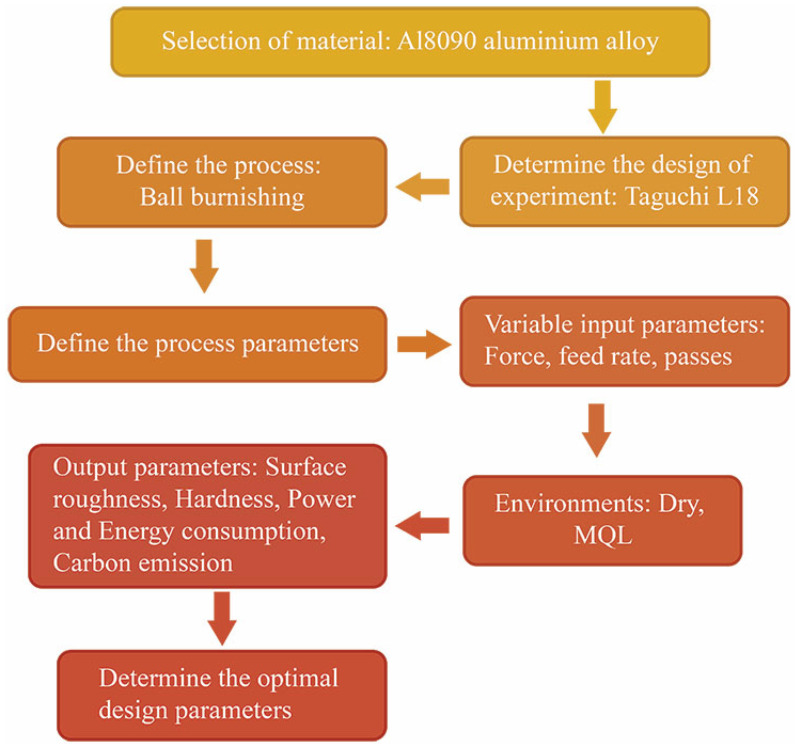
The experimental methodology.

The experimental design methodology used the Taguchi technique to optimize parameters in a systematic manner (Figure 3) [29,30]. This statistical approach was chosen because it is efficient in experimental design and allows for the simultaneous analysis of the effects of several factors [31]. Based on the parameters and their levels, an L_18_ orthogonal array was chosen as the experimental framework, allowing for a balanced examination while reducing the number of experimental trials needed [29]. The L_18_ orthogonal array was selected for this study due to its ability to efficiently handle multiple factors and levels while minimizing the number of experimental runs. This design allows for the evaluation of up to 8 factors with 2 or 3 levels, which aligns well with the factors and levels considered in this study. The L_18_ array ensures a balanced experimental design, where each factor’s influence can be independently assessed without requiring a full factorial design, which would have been resource-intensive. Additionally, the degrees of freedom provided by the L_18_ array were sufficient to analyze the main effects of the selected factors while also allowing for the evaluation of some interactions. This approach has been successfully applied in previous research on burnishing and similar surface treatment processes, demonstrating its suitability for studies involving multiple process parameters [32,33]. The use of the L_18_ array also facilitates a robust mathematical analysis, ensuring that the experimental results are statistically reliable and interpretable [34].

The investigation focused on four key variable parameters: burnishing conditions (dry and MQL), force, feed rate, and the number of passes. These parameters were systematically varied to evaluate their individual and combined effects on surface quality [30]. Surface roughness measurements (Ra and Rz) were designated as the primary output parameters to assess the surface enhancement process’s effectiveness quantitatively.

This study used the “smaller is better” criterion to reduce surface roughness values, consistent with obtaining higher surface quality [29]. The signal-to-noise (S/N) ratio was used to quantitatively assess the impacts of process parameters while controlling for variability and experimental uncertainty. The S/N ratio for the “smaller is better” trait is represented as follows:

(1) SN=−10 log⁡1N∑i=1nYi2

where Yi represents the individual surface roughness measurements for each trial and n represents the number of observations per trial. This analytical methodology allowed for a full assessment of the impact of process parameters and lubrication conditions on surface quality [31].

The findings of this study offer valuable insights into optimizing ball burnishing parameters for Al8090 aluminum alloy, emphasizing the importance of lubrication and process control in producing better surface finishes. The study uses the Taguchi approach to assure robust and efficient optimization, contributing to the progress of precise production procedures for aerospace-grade aluminum alloys.

### 2.4. Surface Quality Evaluation Methods

Surface quality was assessed using a complete measurement and analysis of surface topography factors. Surface roughness was measured using a calibrated Mitutoyo Surftest SJ-210 profilometer (Mitutoyo Corporation, Kawasaki, Japan) and a diamond stylus with a 2 µm tip radius and 60° angle. The measurement technique followed stringent criteria, with the profilometer set to operate with a detection force of 0.75 mN by JIS’01 requirements. The instrument was configured to use a cut-off length of 0.8 mm and an evaluation length of 4 mm while maintaining a constant measurement speed of 0.75 mm/s and Gaussian filtering for efficient signal processing.

Measurements were taken under controlled environmental conditions (22.5 ± 1 °C) to ensure stability and repeatability. Five independent measurements were obtained at strategically chosen points on each specimen, each perpendicular to the burnishing direction, to capture accurate surface profile characteristics. The final surface roughness values were derived as the arithmetic mean of these measurements, with standard deviations (SD) recorded to ensure measurement consistency [35].

Four essential surface roughness metrics were chosen for quantitative assessment: arithmetic average roughness (R_a_), maximum profile height (R_z_), skewness (R_sk_), and mean spacing of profile elements (RSm) [36,37]. The R_a_ parameter, the most commonly used indicator of surface quality, represents the arithmetic mean of the absolute deviations from the mean line over the sampling length. The R_z_ parameter provides additional information by measuring the average maximum height of the profile, offering insight into the peak-to-valley characteristics of the burnished surface. To better characterize the shape and nature of the surface profile, skewness (R_sk_) and the mean spacing of profile elements (R_Sm_) were also analyzed. R_sk_ is a measure of the asymmetry of the profile about the mean line, providing information about the predominance of peaks (positive R_sk_) or valleys (negative R_sk_) on the surface [36,37]. This parameter is particularly valuable for evaluating the functional performance of burnished surfaces, as negative skewness typically indicates better load-bearing capacity and wear resistance. R_Sm_ represents the mean spacing between profile peaks, offering insights into the spatial distribution of surface features and the texture pattern created by the burnishing process. Together, these four parameters provide a comprehensive understanding of both the amplitude and spatial characteristics of the burnished surface.

**Figure 4:**
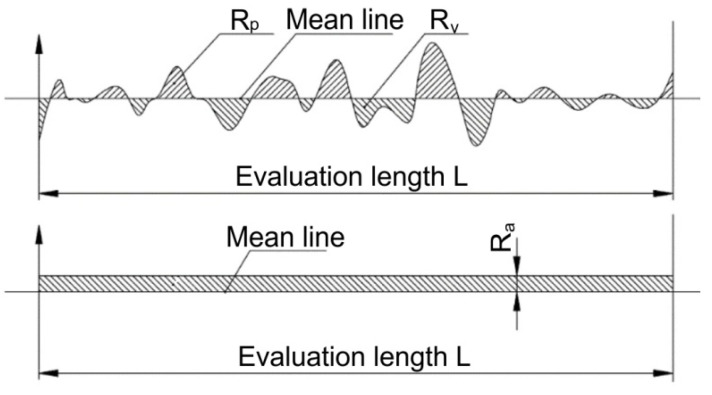
Schematic representation of surface roughness [39].

The mathematical expression for calculating the Ra value, which represents the arithmetic mean of the absolute ordinate values within the sampling length, is given by:

(2) $$R_{a}=\frac{1}{L}\int \left|y\left(x\right)\right|\mathrm{d}x$$

where L represents the sampling length and y(x) denotes the profile height at position x [26]. The R_z_ parameter, which provides insight into the extreme profile characteristics, is determined by [38]:

(3) $$R_{z}=\frac{\sum \left(R_{P}+R_{v}\right)}{n}$$

where R_p_ indicates the maximum peak height, R_v_ represents the maximum valley depth, and n denotes the sampling durations. These mathematical formulas give a quantitative basis for evaluating surface roughness and allow for an exact assessment of the surface quality produced throughout the ball burnishing process (Figure 4) [35,38,39].

The skewness parameter (R_sk_) is calculated using the following equation:

(4) $$R_{sk}=\frac{1}{R_{q}^{3}}.\;\frac{1}{L}\int y^{3}(x)\mathrm{d}x$$

where R_q_ is the root mean square roughness. A positive R_sk_ value indicates a surface dominated by peaks, while a negative value suggests a surface with predominant valleys.

The mean spacing parameter (R_Sm_) is determined by:

(5) $$R_{Sm}=\frac{1}{m}\;\sum X_{si}$$

where m is the number of profile elements within the sampling length, and X_si_ is the width of each profile element. These mathematical formulations provide a quantitative basis for evaluating surface roughness and allow for a precise assessment of the surface quality produced during the ball burnishing process.

### 2.5. Brinell Hardness

Brinell hardness tests were performed to assess the mechanical characteristics of the Al8090 alloy, following ASTM E10 [40]. The Digirock-RSR multifunctional hardness tester (BMS Bulut Makina Co., Kocaeli, Turkiye) was used for the measurements. Prior to testing, the specimens’ surfaces were thoroughly smoothed and cleaned. During each measurement, a 1/16 inch (1.5875 mm) diameter steel ball was positioned on the specimen with a 10 kg preload, followed by an additional load to achieve a total of 100 kg, which was maintained for 15 s. The applied load and indenter size were selected based on the material properties and experimental conditions. Indentations were made at a minimum of three different locations on each sample, and the resulting indentation diameters were digitally recorded to determine the Brinell hardness value.

### 2.6. Power Consumption

The power consumption during the ball burnishing experiments was determined by measuring the current values using a clamp meter (Sanpa Electronic Devices, Istanbul, Turkiye). The experiments were conducted on a Baoji CS6250B lathe under dry and MQL environments. The clamp meter was used to record the current drawn by the lathe during the burnishing process. Initially, the idle current of the lathe (measured as 8.55 A) was recorded to account for the machine’s baseline power consumption. During the burnishing process, the current values were measured for each experiment, and the net current attributable to the burnishing operation was calculated by subtracting the idle current from the total measured current.

The power consumption was then calculated using the formula [41]:

(6) $$P=\sqrt{3\;}\times \;V\;\times \;I\;\times \;cos(\phi )$$

where P is the power consumption in watts, V is the voltage (380 V), I is the net current in amperes, √3 is fixed in the three-phase systems, and cos (ϕ) is the power factor, taken as 0.85 [42]. The calculated power values were converted to kilowatts for further analysis.

The total energy consumption for each experiment was determined by multiplying the power consumption by the processing time. The processing time was calculated based on the feed rate, burnishing length, and number of passes. The following formula was used [42]:

E = P × t(7)

where E is the energy consumption in kilowatt-hours (kWh), P is the power consumption in kilowatts, and t is the processing time in hours. The experiments were performed with varying parameters, including burnishing force (50–200 N), feed rate (0.05–0.2 mm/rev), and the number of passes (1–4). These parameters influenced the burnishing time and, consequently, the energy consumption.

### 2.7. Carbon Emission Analysis

The carbon emissions from the energy consumption were calculated to evaluate the environmental impact of the burnishing process. The carbon footprint was determined using the following Formula [43]:

Carbon Emission = E × EF(8)

where E is the energy consumption in kWh, and EF is the carbon emission factor, taken as 0.5 kg CO_2_/kWh [44,45]. This emission factor reflects the region’s average carbon intensity of electricity generation. The energy consumption values were multiplied by the emission factor for each experiment to estimate the corresponding carbon emissions. The results were then aggregated to compare the total carbon emissions under dry and MQL environments.

### 2.8. Statistical Analysis

The statistical analysis framework utilized ANOVA through statistical software to evaluate the experimental results, with a significance level (α) of 0.05 and a 95% confidence interval [46]. This approach enabled quantification of each parameter’s influence on the response variables through the calculation of the sum of squares (SS), degrees of freedom (DOF), and determination of F-ratio and *p*-values. For developing predictive models, a general linear model (GLM) approach was used to evaluate the relationship between response variables and independent variables. The regression models were expressed in the general form [47]:

Y = β_0_ + β_1_X_1_ + β₂X_2_ + β_3_X_3_ + β_4_X_4_

where Y represents the response variable (arithmetic average roughness Ra, power consumption PC, or energy consumption EC), β₀ is the intercept of the regression model, β_1_, β_2_, β_3_, and β_4_ are the regression coefficients, and X_1_, X_2_, X_3_, and X_4_ include the coded values for the burnishing environment, force, feed rate, and number of passes. These coded values, as presented in Table 1, were assigned specific codes: C1 and C2 for the burnishing conditions (dry and MQL); F1, F2, and F3 for burnishing force (50 N, 100 N, and 200 N); Fr1, Fr2, and Fr3 for feed rate (0.05 mm/rev, 0.1 mm/rev, and 0.2 mm/rev); and P1, P2, and P3 for the number of passes (one, two, and four). This analytical methodology allowed for a comprehensive assessment of the impact of process parameters and lubrication conditions on surface quality and other response variables.

## 3. Results and Discussion

### 3.1. Analysis of Surface Quality and Parameter Effects

Table 3 presents the effects of different ball burnishing parameters (environment, force, feed rate, and number of passes) on surface roughness (R_a_, R_z_) and Brinell hardness. Regarding surface roughness, the R_a_ and R_z_ values obtained in the MQL environment were generally lower than those in dry conditions, indicating better surface quality. The lowest R_a_ value (0.562 µm) was achieved in the MQL environment with a force of 200 N, a feed rate of 0.05 mm/rev, and four passes. Increasing the number of passes improved surface roughness, but the rate of improvement diminished after 2–4 passes. This saturation effect likely occurs due to strain hardening, where excessive plastic deformation prevents further surface smoothening [10]. Regarding Brinell hardness, higher hardness values were observed in dry conditions. The highest hardness (43.6 HB) was achieved with a force of 100 N, a feed rate of 0.1 mm/rev, and two passes in the dry environment. This can be attributed to more significant plastic deformation and surface hardening in dry conditions.

Numerous studies in the literature have demonstrated the effectiveness of ball burnishing in improving surface quality, mechanical properties, and process sustainability. Hassan and Al-Bsharat [48] investigated the influence of the burnishing process on surface roughness, hardness, and microstructure for non-ferrous metals, reporting significant improvements in surface integrity and hardness due to plastic deformation and compressive residual stresses. Similarly, Revankar et al. [49] analyzed the effects of ball burnishing on titanium alloys, highlighting that higher forces and multiple passes enhance surface quality by smoothing surface irregularities and increasing hardness. These findings emphasize the importance of optimizing process parameters to achieve superior surface finishes.

Nguyen et al. [17] and Rotella et al. [18] explored the role of lubrication environments in burnishing processes, particularly focusing on MQL. Their studies revealed that MQL significantly reduces surface roughness by minimizing friction and improving cooling efficiency, making it a more sustainable alternative to dry burnishing. Additionally, Teimouri et al. [50] demonstrated that ultrasonic-assisted ball burnishing of aluminum alloy further enhances surface properties and induces favorable residual stresses, particularly under optimized force and feed rate conditions.

**Figure 5:**
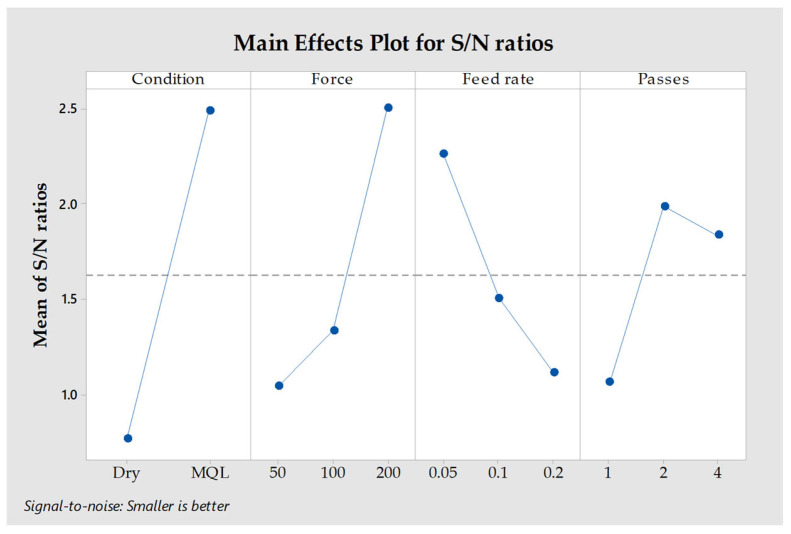
Main effects graph associated with S/N ratios concerning the surface roughness values.

Figure 5 illustrates the main effects of process parameters on the signal-to-noise (S/N) ratio for surface roughness using the “smaller is better” criterion. The MQL environment outperformed dry conditions in reducing surface roughness. Higher forces (200 N) and lower feed rates (0.05 mm/rev) resulted in better surface quality. Additionally, increasing the number of passes improved surface roughness, with two passes being the most effective.

**Figure 6:**
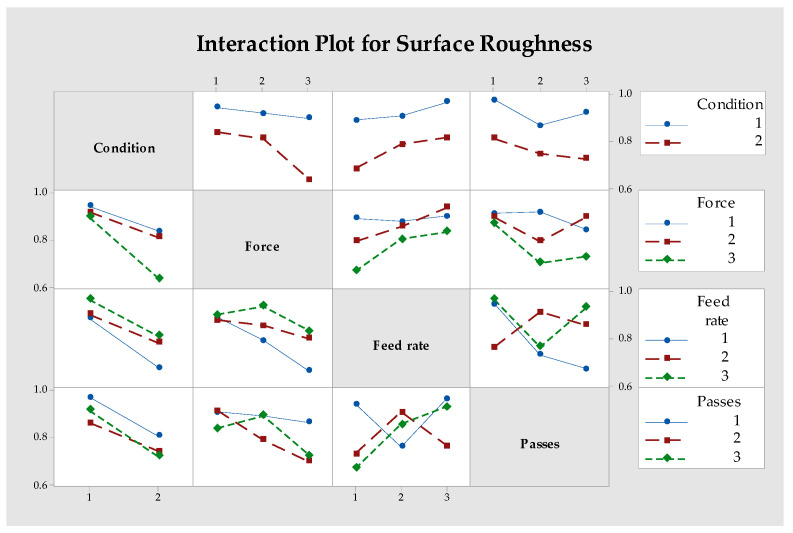
Interaction graph of parameters for S/N ratio evaluated for the surface roughness values.

Figure 6 shows the interaction effects between process parameters on surface roughness. Higher forces combined with lower feed rates significantly improved surface roughness. Similarly, increasing the number of passes amplified the positive effects of higher forces. The combination of lower feed rates and more passes resulted in smoother surfaces.

The results of this study align with the findings of Revankar et al. [49] and Vaishya et al. [11], which indicate that applying higher forces and executing multiple passes significantly enhance the surface quality by smoothing surface irregularities. This suggests that optimizing parameter combinations, rather than focusing on individual parameters, is crucial for achieving the best surface finish outcomes.

**Figure 7:**
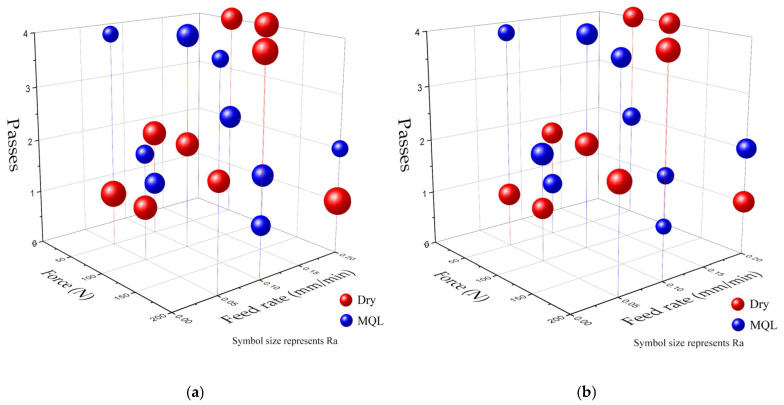
Impact of ball burnishing parameters on (**a**) surface roughness and (**b**) Brinell hardness.

Figure 7 illustrates the effects of ball burnishing parameters on surface roughness and Brinell hardness. For surface roughness, lower values were achieved with higher forces, lower feed rates, and more passes. The MQL environment consistently outperformed dry conditions in reducing surface roughness. For Brinell hardness, higher values were observed with higher forces and fewer passes, particularly in dry conditions. The MQL environment resulted in slightly lower hardness values compared to dry conditions.

Kanovic et al. [12] modeled surface roughness in ball burnishing using regression analysis and artificial neural networks, concluding that higher forces and lower feed rates yield better surface quality. Similarly, Attabi et al. [13] examined the mechanical and wear behaviors of stainless steel following ball burnishing and reported that induced compressive stresses lead to improvements in microhardness and wear resistance. These observations are consistent with the present study, which also demonstrated trade-offs between surface quality and hardness under varying lubrication and process conditions.

### 3.2. Energy Consumption and Environmental Impact Analysis

Table 4 presents the energy consumption and carbon emissions associated with the ball burnishing process under different conditions. The MQL environment resulted in lower energy consumption and carbon emissions than dry conditions. The lowest energy consumption (0.0169 kWh) and carbon emissions (0.0084 kg CO_2_) were observed in the MQL environment with a force of 100 N, a feed rate of 0.2 mm/rev, and one pass. In contrast, dry conditions consume more energy and produce higher carbon emissions due to increased friction and heat generation.

From a sustainability perspective, Makhesana et al. [22] and Sarıkaya et al. [20] emphasized the environmental benefits of MQL, particularly its ability to reduce energy consumption and carbon emissions during machining and surface finishing. Their work underscores the importance of incorporating sustainability metrics, such as power consumption and carbon emissions, into the evaluation of burnishing processes. Furthermore, the adoption of hybrid lubrication techniques not only improves process efficiency by reducing friction but also minimizes the overall environmental impact, reinforcing MQL’s role as a sustainable manufacturing method.

**Figure 8:**
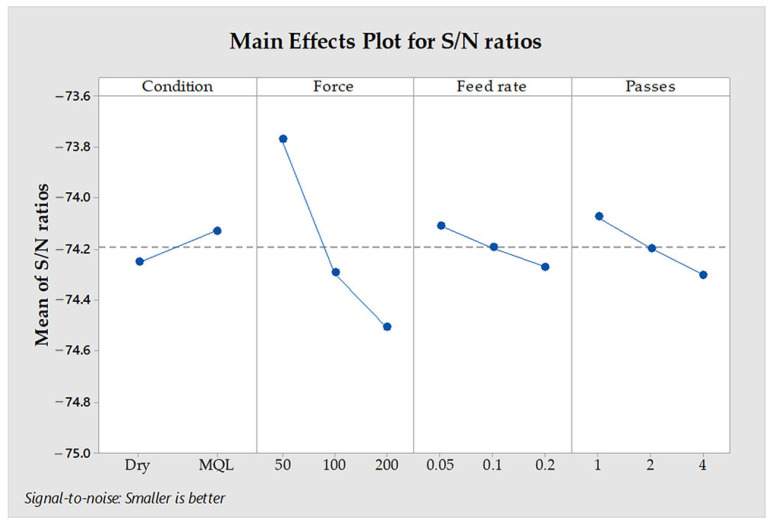
Main effects graph associated with S/N ratios concerning power consumption.

The power consumption data for ball burnishing of Al8090 aluminum–lithium alloy reveals significant influences from various process parameters. Figure 8 presents the main effects plot for S/N ratios related to power consumption, indicating that the most influential factors in descending order are condition > force > number of passes > feed rate.

The MQL condition consistently demonstrates lower power consumption compared to dry conditions. This can be attributed to the superior lubrication properties of MQL, which reduces friction at the tool-workpiece interface [51]. The low-volume oil mist provides adequate lubrication while minimizing resistance during the burnishing process, resulting in reduced power demand from the machine [52].

Regarding the force parameter, a clear trend of increasing power consumption with higher burnishing forces is observed. When examining the data, the highest power consumption values (5193.92–5398.92 W) were recorded at 200 N force. This relationship is expected as higher forces require more power from the machine to maintain the set burnishing pressure [53].

**Figure 9:**
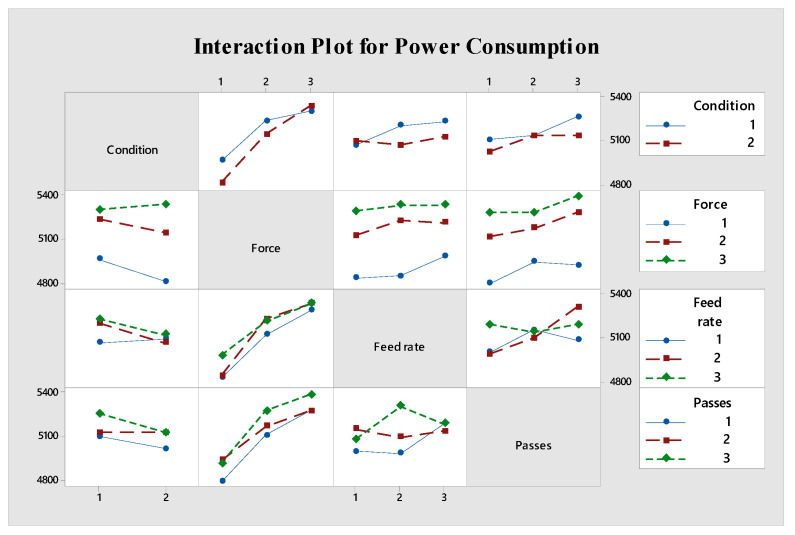
Interaction graph of parameters for S/N ratio evaluated for power consumption.

Figure 9 illustrates the interaction effects between different parameters. A notable interaction exists between the environment and force, where the power consumption increases with force and is more pronounced in dry conditions compared to MQL. This suggests that MQL’s lubricating effect becomes more advantageous at higher forces, mitigating the power increase that would otherwise occur [54].

**Figure 10:**
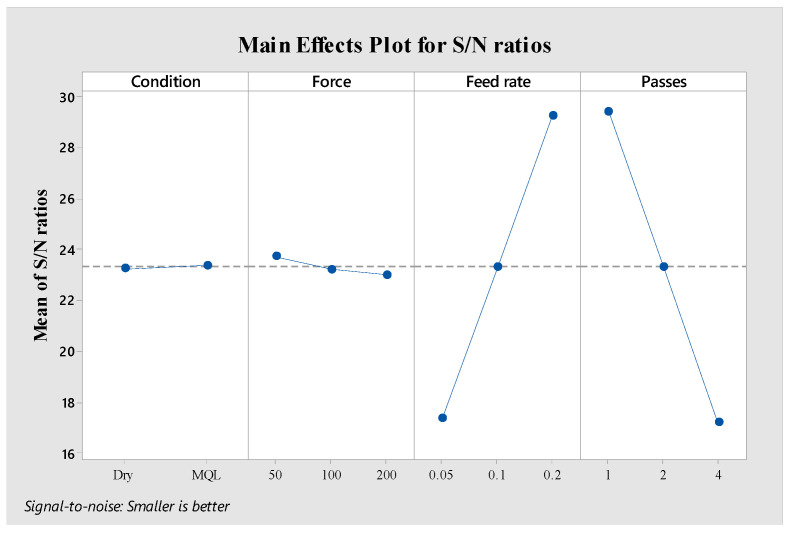
Main effects graph associated with S/N ratios concerning energy consumption.

Energy consumption represents a critical sustainability metric for manufacturing processes. Figure 10 shows the main effects plot for S/N ratios related to energy consumption, revealing that the number of passes is the most significant factor, followed by environment, feed rate, and force.

The MQL environment consistently results in lower energy consumption compared to dry conditions. However, the most dramatic effect comes from the number of passes, with energy consumption increasing substantially as the number of passes increases from 1 to 4. This relationship is directly connected to the processing time, as energy consumption is calculated as power multiplied by time [55].

The lowest energy consumption (0.0169 kWh) was achieved in experiment 15, using the MQL environment with a 100 N force, 0.2 mm/rev feed rate, and a single pass. Conversely, the highest energy consumption (0.2866 kWh) occurred in experiment 16, with the MQL environment, a 200 N force, 0.05 mm/rev feed rate, and four passes.

**Figure 11:**
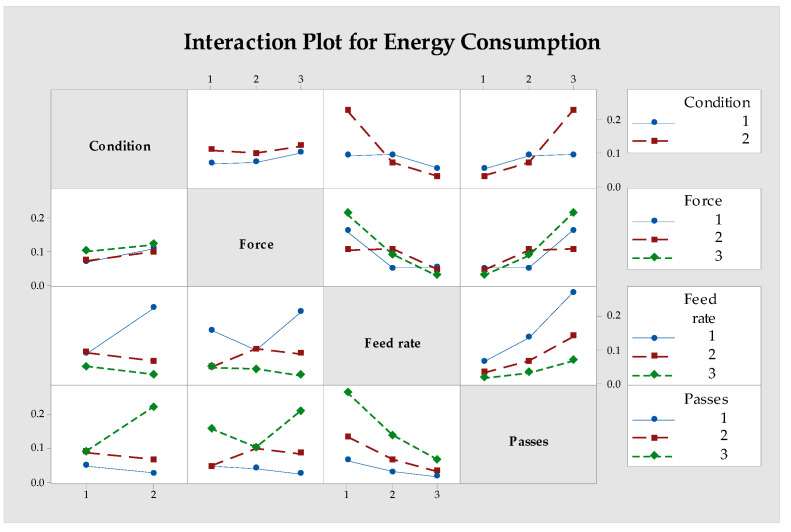
Interaction graph of parameters for S/N ratio evaluated for the energy consumption.

Figure 11 demonstrates important interaction effects, particularly between feed rate and number of passes. At high feed rates (0.2 mm/rev), the effect of increasing passes on energy consumption is less severe than at low feed rates (0.05 mm/rev). This interaction is logical since higher feed rates reduce processing time, which partially offsets the increased energy demand from multiple passes [56].

Carbon emissions follow the same pattern as energy consumption since they are directly calculated based on energy usage with a conversion factor of 0.5 kg CO_2_/kWh [57]. The data indicates that carbon emissions ranged from 0.0084 kg CO_2_ (experiment 15) to 0.1433 kg CO_2_ (experiment 16).

The MQL environment generally results in lower carbon emissions compared to dry conditions under similar parameter settings. This supports the growing emphasis on MQL as an environmentally friendly alternative in modern manufacturing processes [58]. The significant variation in carbon emissions between different parameter combinations (up to 17 times the difference between minimum and maximum values) highlights the importance of parameter optimization from an environmental perspective.

The interaction plots (Figure 9 and Figure 11) reveal complex relationships between parameters. For power consumption, strong interactions exist between environment and force, where MQL shows particular advantages at higher forces. For energy consumption, the interaction between feed rate and number of passes is most notable, with higher feed rates helping to mitigate the energy impact of multiple passes.

The data also shows that certain parameters have divergent effects on different outputs. For instance, while higher forces increase power consumption, they may not proportionally increase energy consumption if combined with higher feed rates that reduce processing time. This demonstrates the need for balanced parameter selection based on specific manufacturing objectives [59].

### 3.3. Statistical Analysis Results

Statistical analysis was used to objectively assess the impact of process factors on response variables and create predictive models for optimization. ANOVA was used to establish the statistical significance of each parameter’s influence on surface roughness, power consumption, and energy consumption. The generated regression equations give mathematical links between the coded process parameters and the measured responses, allowing for the prediction of outcomes for different parameter combinations. The ANOVA results for surface roughness (Ra) are presented in Table 5, power consumption (PC) in Table 6, and energy consumption (EC) in Table 7.

The ANOVA results for surface roughness indicate that the burnishing condition is the most significant factor, with a *p*-value of 0.001, suggesting that the choice between dry and MQL environments significantly affects surface roughness. Force is also statistically significant (*p*-value = 0.027), while feed rate shows marginal significance (*p*-value = 0.068). The number of passes is not statistically significant (*p*-value = 0.112) at the 0.05 significance level. The model explains 82.54% of the total variation in surface roughness (R-sq = 82.54%), indicating a good model fit for the experimental data.

The regression equation for surface roughness is:

Ra = 0.8392 + 0.0796 C1 − 0.0796 C2 + 0.0503 F1 + 0.0230 F2 − 0.0733 F3 − 0.0555 Fr1 + 0.0053 Fr2 + 0.0502 Fr3 + 0.0528 P1 − 0.0353 P2 − 0.0175 P3

For power consumption, the ANOVA results reveal that force is the most influential parameter (*p*-value < 0.001), followed by number of passes (*p*-value = 0.017) and burnishing condition (*p*-value = 0.047). The feed rate shows marginal significance with a *p*-value of 0.089. The statistical model for power consumption demonstrates an excellent fit with an R-squared value of 94.17%, suggesting that the model effectively captures the relationships between the process parameters and power consumption.

The regression equation for power consumption is:

PC = 5128.3 + 35.0 C1 − 35.0 C2 − 244.9 F1 + 57.7 F2 + 187.1 F3 − 48.9 Fr1 + 3.8 Fr2 + 45.1 Fr3 − 67.7 P1 + 1.8 P2 + 65.9 P3

The ANOVA results for energy consumption show that feed rate and number of passes are the most significant factors (both with *p*-values < 0.001), followed by burnishing condition (*p*-value = 0.036). Interestingly, force does not exhibit statistical significance (*p*-value = 0.206) for energy consumption. The model explains 93.61% of the variation in energy consumption data (R-sq = 93.61%), indicating excellent model performance.

The regression equation for energy consumption is:

EC = 0.09313 − 0.01459 C1 + 0.01459 C2 − 0.00682 F1 − 0.00950 F2 + 0.01632 F3 + 0.06522 Fr1 − 0.01235 Fr2 − 0.05287 Fr3 − 0.05403 P1 − 0.01325 P2 + 0.06728 P3

### 3.4. Limitations and Future Research Directions

While this study provides valuable insights into optimizing ball burnishing parameters for Al8090 aluminum–lithium alloy, several important considerations must be acknowledged regarding practical applications in aerospace environments. Despite the promising surface quality improvements achieved through ball burnishing, aluminum–lithium alloys exhibit significant environmental sensitivity that may affect long-term performance. These alloys are particularly susceptible to environmentally assisted cracking, stress corrosion cracking, and exfoliation corrosion when exposed to moisture and corrosive environments [2,60]. This environmental sensitivity could potentially compromise the material’s longevity and negate surface enhancements achieved through burnishing processes.

In aerospace applications, aluminum components typically undergo anodization treatments to mitigate environmental effects [61]. However, this protective process introduces surface-breaking material defects that can significantly reduce component durability and serve as initiation sites for fatigue cracks under cyclic loading conditions [1]. The present study did not investigate the interaction between ball burnishing and subsequent anodization processes, which represents an important area for future research.

Another critical limitation relates to fastener holes, which are fundamental to aerospace structures. Molent and Barter [62] and Wanhill [63] demonstrated that the operational life of aerospace structures made from Al-Li alloys is frequently determined by the nucleation and growth of small three-dimensional cracks (typically around 0.01 mm) that develop along the bore of fastener holes. Since these holes are generally drilled after anodization or other surface treatments, they represent untreated regions that cannot benefit from burnishing processes.

Based on these limitations, future research should investigate (1) the long-term effects of ball burnishing on the environmental resistance of Al8090, (2) the interaction between burnishing and subsequent anodization processes, and (3) specialized techniques to enhance the fatigue resistance of fastener holes. These research directions would provide a more complete understanding of the practical benefits of ball burnishing for Al8090 components in aerospace applications.

## 4. Conclusions

This study systematically evaluated the effects of ball burnishing parameters on the surface quality, mechanical properties, and environmental impact of Al8090 aluminum–lithium alloy. The findings demonstrated that the MQL environment consistently outperformed dry conditions in reducing surface roughness, achieving the lowest R_a_ value of 0.562 µm with a force of 200 N, a feed rate of 0.05 mm/rev, and four passes. This improvement is attributed to the superior lubrication and cooling properties of MQL, which reduce friction and enhance surface integrity. In contrast, the highest Brinell hardness (43.6 HB) was observed in dry conditions with a force of 100 N, a feed rate of 0.1 mm/rev, and two passes, likely due to increased plastic deformation and strain hardening without lubrication.

Energy consumption and carbon emissions were also analyzed, revealing that the MQL environment significantly reduced environmental impact. The lowest energy consumption (0.0169 kWh) and carbon emissions (0.0084 kg CO_2_) were recorded with a force of 100 N, a feed rate of 0.2 mm/rev, and one pass in the MQL environment. These results highlight the environmental benefits of MQL, which aligns with modern sustainable manufacturing practices. However, while less environmentally friendly, dry conditions produced higher hardness values, indicating a trade-off between surface quality, mechanical properties, and sustainability.

The findings of this study have significant practical implications, particularly for industries such as aerospace and automotive, where high-performance and lightweight materials like Al8090 are critical. The results demonstrate that the MQL environment not only enhances surface quality but also reduces energy consumption and carbon emissions, making it a viable option for sustainable manufacturing. These insights provide a pathway for optimizing ball burnishing processes to achieve both technical and environmental objectives.

The study also confirmed that the Taguchi L_18_ design is an effective method for optimizing process parameters, allowing for a systematic evaluation of individual and combined effects. The results emphasize the importance of balancing process parameters to achieve desired outcomes, such as superior surface quality, enhanced mechanical properties, and reduced environmental impact.

Although this study gives useful insights into improving ball burnishing parameters for Al8090 aluminum–lithium alloy, it should be viewed as a preliminary examination that demonstrates the possibility for balancing process parameters to obtain desired results. In experimental conditions, the findings indicate promise for improving surface quality, mechanical properties, and reducing environmental impact. However, more research is needed to determine the long-term effectiveness of these optimized parameters in operational aerospace applications, especially given the environmental sensitivity of Al-Li alloys, the effects of subsequent anodization processes, and the critical role of fastener holes in determining the service life of aerospace structures.

## Figures and Tables

**Table 1:** Chemical composition and physical properties of Al8090 aluminum alloy.

**Chemical Composition** **Elements in wt. %**	Al: 94.7, Li: 2.5, Cu: 1.3, Mg: 0.9, Fe: 0.15, Zn: 0.15, Si: 0.05, Ti: 0.05, Cr: 0.05, Mn: 0.05, Zr: 0.1
**Physical properties**	
Tensile strength (MPa)	450
Yield strength (MPa)	370
Hardness (Brinell)	38
Elongation at break (%)	7
Density g/cm^3^	2.54

**Table 2:** Process parameters and their limits [28].

Factors		Levels		
	Code	1	2	3
Conditions	C	Dry	MQL	-
Force (N)	F	50	100	200
Feed rate (mm/rev)	Fr	0.05	0.1	0.2
Passes	P	1	2	4

**Table 3:** Parameters and results after the burnishing process.

Exp.			Parameters											
	Condition	Force (N)	Feed Rate (mm/rev)	Passes	Ra (µm)	SD	Rz (µm)	SD	Rsk -	SD	**Rsm (µm)**	**SD**	**Hardness** **HB**	**SD**
1	Dry	50	0.05	1	0.999	0.086	4.550	0.317	−0.135	0.025	183.3	12.3	40.4	2.76
2	Dry	50	0.1	2	0.933	0.074	3.977	0.278	0.081	0.032	100.4	8.6	40.2	1.61
3	Dry	50	0.2	4	0.898	0.88	5.080	0.501	0.152	0.017	233.1	16.3	40.1	1.95
4	Dry	100	0.05	1	0.891	0.102	3.796	0.370	−0.106	0.028	196.8	12.7	38.4	2.75
5	Dry	100	0.1	2	0.888	0.701	4.267	0.393	−0.120	0.021	211.0	7.8	43.6	3.04
6	Dry	100	0.2	4	0.968	0.652	4.334	0.277	−0.267	0.037	203.2	14.1	40.0	2.74
7	Dry	200	0.05	2	0.769	0.112	3.972	0.345	−0.223	0.019	293.8	10.6	41.6	2.55
8	Dry	200	0.1	4	0.893	0.093	4.093	0.402	−0.324	0.027	152.7	14.9	40.4	2.41
9	Dry	200	0.2	1	1.030	0.071	4.911	0.298	−0.172	0.021	201.4	11.5	39.2	1.86
10	MQL	50	0.05	4	0.784	0.086	4.035	0.416	−0.566	0.031	251.1	16.8	39.0	2.27
11	MQL	50	0.1	1	0.821	0.070	3.564	0.341	0.164	0.015	358.0	18.5	37.8	2.66
12	MQL	50	0.2	2	0.902	0.156	4.629	0.662	−0.044	0.018	241.0	14.7	37.4	3.25
13	MQL	100	0.05	2	0.697	0.048	3.193	0.337	0.025	0.125	166.3	10.3	40.8	2.83
14	MQL	100	0.1	4	0.825	0.082	4.152	0.531	0.012	0.022	150.9	18.4	38.8	1.72
15	MQL	100	0.2	1	0.904	0.051	4.281	0.386	0.307	0.026	268.9	17.2	34.0	2.89
16	MQL	200	0.05	4	0.562	0.069	2.756	0.305	−0.142	0.019	190.5	9.6	32.5	3.46
17	MQL	200	0.1	1	0.707	0.062	3.524	0.451	−0.267	0.034	298.0	19.3	27.7	3.35
18	MQL	200	0.2	2	0.634	0.083	2.951	0.293	−0.128	0.011	200.8	12.1	36.6	2.97

**Table 4:** Power and energy consumption and carbon emissions in the ball burnishing process.

Exp.	Conditions	Force (N)	Feed Rate (mm/rev)	Passes	Power Consumption (W)	Energy Consumption (kWh)	Carbon Emission (kg CO_2_)
1	Dry	50	0.05	1	4867.88	0.0649	0.0324
2	Dry	50	0.1	2	4965.89	0.0662	0.0331
3	Dry	50	0.2	4	5041.92	0.0672	0.0336
4	Dry	100	0.05	1	5135.68	0.0685	0.0343
5	Dry	100	0.1	2	5230.89	0.0697	0.0349
6	Dry	100	0.2	4	5336.68	0.0712	0.0356
7	Dry	200	0.05	2	5193.92	0.1385	0.0693
8	Dry	200	0.1	4	5398.92	0.1430	0.0715
9	Dry	200	0.2	1	5297.92	0.0177	0.0089
10	MQL	50	0.05	4	4789.92	0.2553	0.1277
11	MQL	50	0.1	1	4718.92	0.0315	0.0158
12	MQL	50	0.2	2	4915.92	0.0328	0.0164
13	MQL	100	0.05	2	5109.92	0.1363	0.0682
14	MQL	100	0.1	4	5218.92	0.1392	0.0696
15	MQL	100	0.2	1	5083.92	0.0169	0.0084
16	MQL	200	0.05	4	5378.92	0.2866	0.1433
17	MQL	200	0.1	1	5258.92	0.0351	0.0176
18	MQL	200	0.2	2	5363.92	0.0358	0.0179

**Table 5:** Details of ANOVA results of surface roughness for the S/N ratio.

Parameters	DOF	SS	MS	F-Value	*p*-Value
Condition	1	0.11408	0.114083	24.02	0.001
Force (N)	2	0.05064	0.025321	5.33	0.027
Feed rate (mm/rev)	2	0.03375	0.016876	3.55	0.068
Passes	2	0.02608	0.013038	2.75	0.112
Error	10	0.04749	0.004749		
Total	17	0.27204			

**Table 6:** Details of ANOVA results of power consumption for the S/N ratio.

Parameters	DOF	SS	MS	F-Value	*p*-Value
Condition	1	22,079	22,079	5.16	0.047
Force (N)	2	589,892	29,4946	68.87	0.000
Feed rate (mm/rev)	2	26,642	13,321	3.11	0.089
Passes	2	53,635	26,817	6.26	0.017
Error	10	42,829	4283		
Total	17	735,077			

**Table 7:** Details of ANOVA results of energy consumption for the S/N ratio.

Parameters	DOF	SS	MS	F-Value	*p*-Value
Condition	1	0.003831	0.003831	5.89	0.036
Force (N)	2	0.002418	0.001209	1.86	0.206
Feed rate (mm/rev)	2	0.043204	0.021602	33.24	0.000
Passes	2	0.045733	0.022867	35.18	0.000
Error	10	0.006499	0.000650		
Total	17	0.101685			

## Data Availability

The original contributions presented in this study are included in the article. Further inquiries can be directed to the corresponding author.
